# Effect of Boron Doping on the Interlayer Spacing of Graphite

**DOI:** 10.3390/ma15124203

**Published:** 2022-06-13

**Authors:** Chenguang Bao, Qing Zeng, Fujin Li, Lei Shi, Wei Wu, Li Yang, Yuxi Chen, Hongbo Liu

**Affiliations:** 1 College of Materials Science and Engineering, Hunan University, Changsha 410082, China; 18650765569@163.com (C.B.); hunanyangli@aliyun.com (L.Y.); 2 Hunan Provincial Key Laboratory of Flexible Electronic Materials Genome Engineering, School of Physics and Electronic Sciences, Changsha University of Science and Technology, Changsha 410114, China; zengqing@stu.csust.edu.cn; 3 College of Materials Science and Engineering, Changsha University of Science & Technology, Changsha 410114, China; lifujin511@163.com; 4 Hunan Zhongke Shinzoom Co., Ltd., Changsha 410118, China; lshi@shinzoom.com (L.S.); wuwei202206@163.com (W.W.)

**Keywords:** boron-doped graphite, substitutional boron, interlayer spacing, DFT calculations

## Abstract

Boron-doped graphite was prepared by the heat treatment of coke using B_4_C powder as a graphitization catalyst to investigate the effects of the substitutional boron atoms on the interlayer spacing of graphite. Boron atoms can be successfully incorporated into the lattice of graphite by heat treatment, resulting in a reduction in the interlayer spacing of graphite to a value close to that of ideal graphite (0.3354 nm). With an increase in the catalyst mass ratio, the content of substituted boron in the samples increased significantly, causing a decrease in the interlayer spacing of the boron-doped graphite. Density functional theory calculations suggested that the effects of the substitutional boron atoms on the interlayer spacing of the graphite may be attributed to the transfer of Π electrons between layers, the increase in the electrostatic surface potential of the carbon layer due to the electron-deficient nature of boron atoms, and Poisson contraction along the c-axis.

## 1. Introduction

Boron has been widely used as a graphitization catalyst during the preparation of carbonaceous materials for application in lithium-ion battery anodes because it can reduce the graphitization temperature and promote the degree of graphitization [1,2,3,4]. As is well known, boron atoms can be inserted into graphite to form B_4_C clusters. In addition, some boron atoms can substitute for the carbon atoms, since the atomic radius of boron is close to that of carbon, which can effectively influence the structure, electrochemical performance and physicochemical properties of graphite [5,6,7,8,9]. One prominent structural characteristic of boron-doped carbon materials is the obvious decrease in the interlayer spacing. Many researchers have reported that boron-doped graphite has a smaller interlayer spacing than that of ideal graphite (0.3354 nm) [10,11,12].

A decrease in the interlayer spacing of boron-doped graphite is considered to be associated with dissolution–precipitation of the carbon atoms and the substitutional boron atoms (sub-B) in the lattice, while boron atoms occupying the interstitial positions enlarge the interlayer spacing [10,12,13]. The carbon dissolution–precipitation mechanism is widely referenced to understand the excellent catalytic effects of boron, which can accelerate the gradual homogeneous graphitization of graphite. However, it is difficult to understand the smaller interlayer spacing of boron-doped graphite compared to ideal graphite. There are two possible mechanisms for the effect of sub-B on the interlayer spacing of graphite: (1) the decrease in d_002_ upon boron doping may be related to the depleted p-electrons between the graphitic layers, which leads to a shorter interlayer distance and a lower density of Π-electrons within the graphite layers because of the lower valence state of boron relative to carbon [1]; (2) the decrease in the interlayer spacing is mainly caused by Poisson contraction along the c-axis due to the expansion along the a-axis. On the contrary, the depleted p-electrons between graphitic layers by sub-B result in an enlargement of the interlayer spacing [14]. To date, as far as we are aware, no further theoretical study has been conducted and no general understanding has been reached as to the effect of sub-B on the interlayer spacing of graphite.

In this paper, needle coke and pitch coke were chosen as raw materials, and B_4_C powder was used as a graphitization catalyst to prepare boron-doped graphite. The variation in the sub-B content in the carbon lattice with the addition of the catalyst and the effects of the sub-B on the interlayer spacing of the graphite were systematically investigated. The mechanism of the sub-B-induced decrease in the interlayer spacing was revealed based on the analysis of the partial density of states, Mulliken partial charges, total charge density, electrostatic surface potential and Poisson’s ratio of the graphite using density functional theory (DFT) calculations.

## 2. Experimental Methods

### 2.1. Synthesis of Boron-Doped Graphite

Needle coke and pitch coke powder (SHINZOOM Co., Changsha, China) were chosen as the raw materials, and B_4_C powder (C-W NANO Co., Shanghai, China) was used as graphitization catalyst. To prepare boron-doped graphite, B_4_C powder and coke were homogeneously mixed and then transferred into a graphite crucible, which was placed in the center of a medium-frequency induction graphitizing furnace. The furnace was evacuated and flushed with Ar gas three times to completely remove the oxygen and moisture, avoiding their negative impacts. The temperature of the furnace was raised to 2700 ℃ at a heating rate of 10 ℃/min and annealed for 2 h under an Ar atmosphere. Four mass ratios of B_4_C relative to coke (0%, 3%, 6% and 9%) were chosen to synthesize the products, which were denoted as 0GC27, 3GC27, 6GC27 and 9GC27, respectively, when the needle coke was used as raw material. The samples prepared with different mass ratios of B_4_C relative to pitch coke (0%, 3%, 6% and 9%) were denoted as 0PIC27, 3PIC27, 6PIC27 and 9PIC27, respectively.

### 2.2. Characterization of the Graphitized Samples

X-ray diffraction measurements were carried out on a Brucker D8 X-ray diffractometer using Cu Kα radiation (λ = 0.15406 nm). Silicon was used as an internal standard to calibrate the instrumental error in XRD [15]. The interlayer spacing of the graphite samples was calculated using the Bragg equation.

X-ray photoelectron spectroscopy (XPS, ESCALAB 250Xi) was used to characterize the bonding state of boron in the samples. The total amount of boron with respect to carbon in the samples was estimated by calculating the ratio between the C1s and B1s peak areas and considering sensitivity factors, i.e., boron atoms were assumed to be doped uniformly into the deeper graphite layers.

### 2.3. Models and Computational Details

All the calculations were performed using the DMol3 package of Materials Studio on the basis of DFT [16,17]. The Perdew–Burke–Ernzerhof (PBE) model under the generalized gradient approximation [18] and double-numeric quality basis set with polarization functions were chosen to deal with the electron exchange–correlation interactions. All-electron relativistic calculations were used to treat the core electrons. Linear combination of atomic orbitals was employed to describe the interactions formed between the electrons and ions. Brillouin zone integrations were performed with a 5 × 5 × 1 Monkhorst–Pack k-point mesh to calculate total energies. To optimize the geometric structure, the energy convergence standard of the system was set to 1 × 10^−5^ Ha, atomic force < 0.02 Ha/Å, and maximum ion displacement 0.005 Å for the calculations. Because the weak interactions were not well-described by the standard PBE functional, the empirical dispersion-corrected density functional theory approach proposed by Grimme [19,20] was used to describe the interactions between model layers.

In order to reveal the mechanism of the effect of the substituted boron atoms on the interlayer spacing in the graphite lattice, the corresponding periodic boron-doped graphite (ABA stacking) supercell structure was established. The mass ratio of boron was set at ~1.78% based on the results obtained from our experiments. Two kinds of boron-doped graphite models were optimized and the calculations of the partial density of states, Mulliken partial charges, total charge density, electrostatic surface potential and Poisson’s ratio were based on the optimized structure.

## 3. Results and Discussion

### 3.1. Characterization of Boron-Doped Graphite

**Figure 1:**
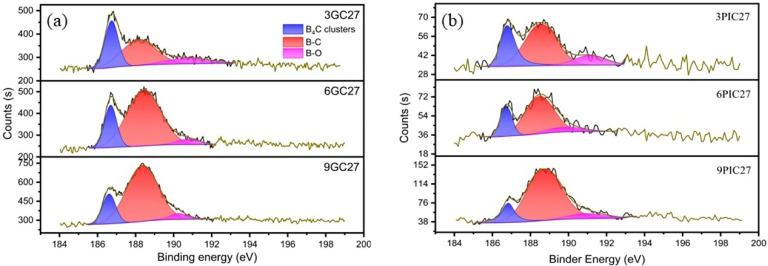
B1s XPS spectra of the boron-doped graphite studied. (**a**) Boron-doped graphite prepared using needle coke (GC) as raw material; (**b**) Boron-doped graphite prepared using pitch coke (PIC) as raw material.

**Figure 2:**
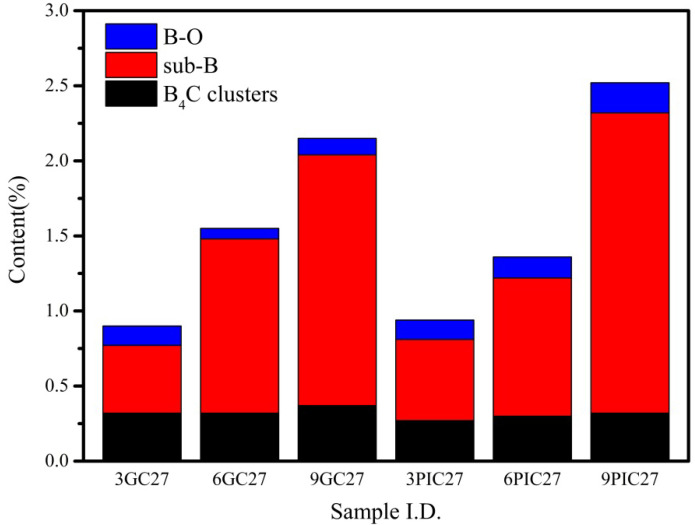
Content of the boron states in the samples, obtained using B1s XPS spectra. This chart is a visualization of the data in Table 1.

Boron-doped graphite was successfully prepared using needle coke and pitch coke as raw materials and B_4_C powder as the graphitization catalyst. The states of boron in the samples were characterized using XPS, and the XPS B1s core-level spectra of the samples as well as their deconvolution are depicted in Figure 1. For the 3GC27, 6GC27, 9GC27, 3PIC27, 6PIC27 and 9PIC27 samples, the B1s band could be deconvoluted into three binding-energy peaks at 186.9, 188.7 and 190.3 eV, corresponding to the B atoms in the B_4_C clusters, the sub-B structure and the O–B bonding structure, respectively [21,22]. The total amounts of boron with respect to carbon in the 3GC27, 6GC27, 9GC27, 3PIC27, 6PIC27 and 9PIC27 samples were 0.9%, 1.55%, 2.15%, 0.94%, 1.36% and 2.52%, respectively, as listed in Table 1; the amounts were estimated by calculating the ratio of C1s to B1s. To better visualize these results, Figure 2 shows the percentiles of each boron state according to Table 1. For the samples prepared using needle coke as the raw material, the content of B_4_C clusters and B-O did not vary much with the addition of a greater amount of catalyst, whereas the amount of sub-B significantly increased as the mass ratio of the catalyst increased. Furthermore, the same phenomenon was discovered when pitch coke was used as the raw material as shown in Figure 2, suggesting that an adjustment to the amount of catalyst most substantially impacted the sub-B content.

**Figure 3:**
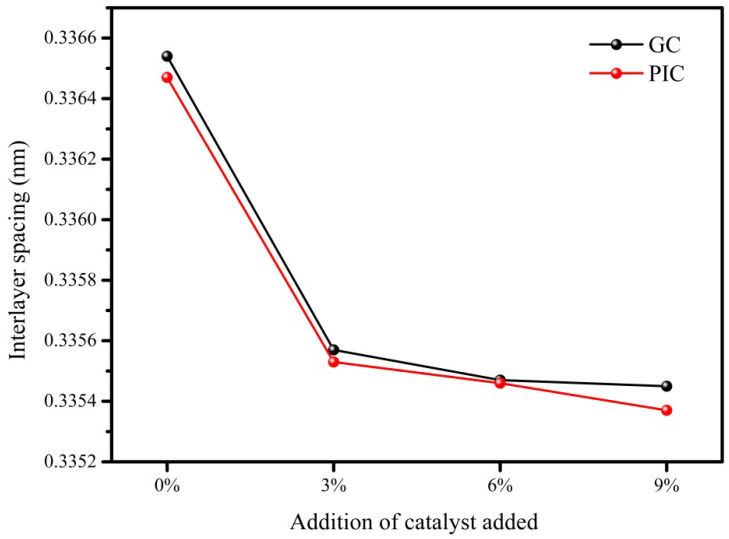
Interlayer spacings of the samples as a function of the amount of catalyst added.

The interlayer spacings of the samples were calculated using XRD and the Bragg equation and are listed in Table 2. Moreover, the interlayer spacings of the samples as a function of the amount of catalyst used are displayed in Figure 3. For the samples prepared using needle coke and pitch coke, the interlayer spacings of the samples heated to 2700 ℃ apparently decreased with an increase in the amount of catalyst up to 3%, then slightly decreased upon further increasing the catalyst loading, revealing that boron doping can significantly improve the degree of graphitization [23,24]. The interlayer spacing of 9GC27 and 9PIC27 were 0.33545 nm and 0.33537, respectively. It is worth noting that the interlayer spacing of 9PIC27 was slightly lower than that of ideal graphite (0.3354 nm). When combined with the results obtained from the XPS analysis indicating the obvious increase in sub-B content in the boron-doped graphite samples after adjusting the amount of catalyst, it can be deduced that the decrease in the interlayer spacing of the samples was associated with the increase in the sub-B content. Sub-B atoms play an important role in decreasing the interlayer spacing of graphite, accounting for the fact that the interlayer spacings of some boron-doped graphite samples were lower than that of ideal graphite (0.3354 nm) [11].

### 3.2. Effect of Substitutional Boron on the Interlayer Spacing

**Figure 4:**
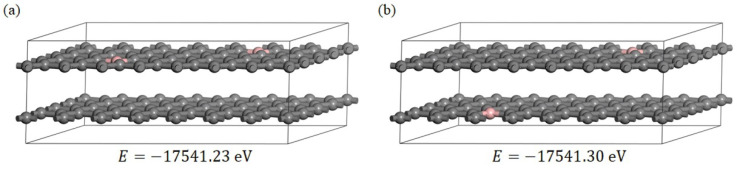
(**a**,**b**) two boron-doped graphite models, in which the pink atoms are boron and the grey atoms are carbon; E is the total energy of the models after optimization of the geometry.

Two boron-doped graphite models were established, as shown in Figure 4a,b, to reveal the effect of sub-B on the interlayer spacing of graphite. The value of E in Figure 4 presents the total energy of each boron-doped graphite model after optimizing the geometry. It can be seen that the total energy of boron-doped graphite model (b) after geometry optimization was the lowest, indicating that the structure in model (b) was the most stable. The interlayer spacings calculated for pristine graphite and model (b) are listed in Table 3. The interlayer spacing of model (b) was 0.3323 nm, lower than that of 9GC27 (0.33545 nm) and 9PIC27 (0.33537 nm). Although the interlayer spacing of model (b) did not match the experimental results, the interlayer spacing calculated for the graphite decreased after boron doping, which was consistent with the variations in interlayer spacing observed in the experiment. Therefore, model (b) was used to further investigate the effect of the Poisson contraction on the interlayer spacing using Poisson’s ratio and the effect of the electronic structure of boron-doped graphite on the interlayer spacing using the partial density of states, Mulliken partial charges, total charge density and electrostatic surface potentials.

#### 3.2.1. The Effect of Poisson Contraction on the Interlayer Spacing

The decrease in the lattice parameter (c) caused by Poisson contraction along the c-axis ${\left(\Delta c_{0}\right)}_{p}$ can be expressed as follows [14]:

(1) $${\left(\Delta c_{0}\right)}_{p}=-3\sqrt{3}\left(d_{C-B}-d_{C-C}\right)\left(\frac{c_{0,G}}{a_{0,G}}\right)\left(-\frac{s_{13}}{s_{11}}\right)\cdot \;x_{B}$$

where $a_{0,G}$ and $c_{0,G}$ are the lattice parameters of the non-doped graphite sample; $d_{C-B}$ and $d_{C-C}$ correspond to the bond length of C–B and C–C, respectively; $x_{B}$ is the atomic fraction of boron in model (b); $s_{13}$ and $s_{11}$ are the components of the elastic compliance tensor; and $-s_{13}/s_{11}$ is the Poisson’s ratio. The values of $a_{0,G}$, $c_{0,G}$, $d_{C-B}$, $d_{C-C}$, $s_{11}$ and $s_{13}$ were obtained from the optimized boron-doped graphite model (b) and are listed in Table 4, in which the bond length is the mean value of the length of the B–C or C–C bonds at different locations. Hence, the variable value of lattice parameter  c derived from Poisson contraction, ${\left(\Delta c_{0}\right)}_{p}$, was calculated to be −0.0003 nm.

The decrease in the lattice parameter  c of graphite caused by boron doping $\left(\Delta_{c}\right)$ can be expressed using the following equation:

(2) $$\Delta c=c_{B,G}-c_{0,G}$$

where $c_{B,G}$ is the lattice parameter of the boron-doped graphite. The value of $c_{B,G}$ obtained from the optimized boron-doped graphite model (b) was 0.6646 nm. Therefore, the $\Delta_{c}$ value calculated from Equation (2) was −0.0092 nm, which is significantly lower than ${\left(\Delta c_{0}\right)}_{p}$ (−0.0003 nm), indicating that the decrease in the interlayer spacing caused by boron doping was much larger than that derived from Poisson contraction. Poisson contraction contributes less to the decrease in the interlayer spacing.

#### 3.2.2. The Electronic Structure of the Boron-Doped Graphite

**Figure 5:**
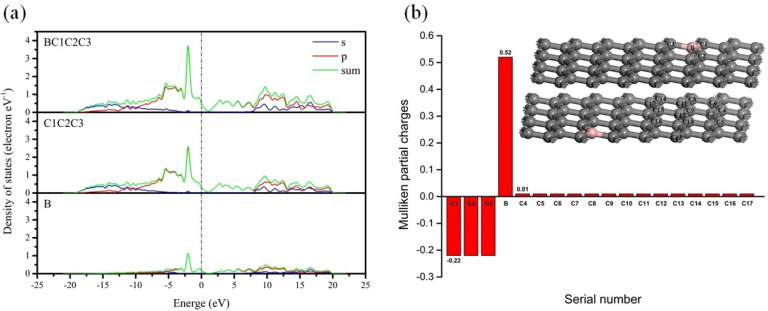
(**a**) Partial density of states of B, C1C2C3 and BC1C2C3. (**b**) Mulliken partial charges of some atoms in model (**b**). Inset: the serial numbers of the atoms in the layers of model (**b**). Carbon: dark grey; boron: pink.

Figure 5a exhibits the partial density of states (PDOS) of B, C1C2C3 and BC1C2C3, labeled in Figure 5b, where the dotted lines represent the Fermi level (E_F_). As shown in Figure 5a, the p orbitals of boron and carbon atoms in model (b) showed obvious hybridization phenomena around −2.4 eV, indicating a strong interaction between the boron and carbon atoms. The Mulliken partial charges of the boron-doped graphite samples were calculated to study the charge transfer between the atoms in boron-doped graphite, as displayed in Figure 5b. It is clear that the charge was mainly transferred from the boron atoms bearing partial positive charges to the nearest neighboring carbon atoms bearing partial negative charges after boron doping, which can be attributed to the higher electronegativity of carbon with respect to boron. Moreover, the total value of the Mulliken partial charges around the boron atoms was −0.14, while that of the carbon atoms in the adjacent carbon layer near the boron atoms was 0.14, implying the presence of electron transfer between the layers around the boron atoms. The same phenomenon was also found in model (a) (Supplementary Figure S1, see details in Supplementary Materials).

**Figure 6:**
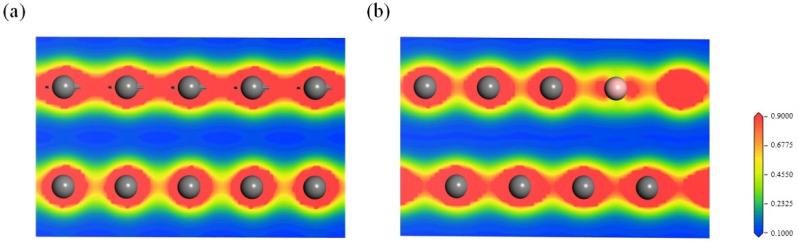
Charge density diagram within the (010) plane of (**a**) graphite and (**b**) boron-doped graphite across the boron atoms. Carbon: dark grey, boron: pink.

Figure 6 depicts the charge density of graphite and boron-doped graphite along the (010) crystal plane, which clearly shows the charge cloud distributed around the atoms. The red and blue regions correspond to the regions with high and low electron density, respectively. Figure 6a shows that the Π electron clouds between the layers of the graphite partially overlapped, which is consistent with the previous report in [25]. Compared with graphite, the charge density between the layers in the boron-doped graphite varied significantly due to the relative displacement of the layers. However, the Π electron clouds around the boron atoms between the layers of graphite still partially overlapped, indicating that boron doping increased the overlapping of the Π electron clouds between the layers. For model (a), there was no relative displacement between the layers, and the charge density between the layers in the boron-doped graphite was slightly enhanced (Supplementary Figure S2). The increase in the overlap of the Π electron clouds between the layers was the result of electron transfer between the layers and the decreased interlayer spacing.

**Figure 7:**
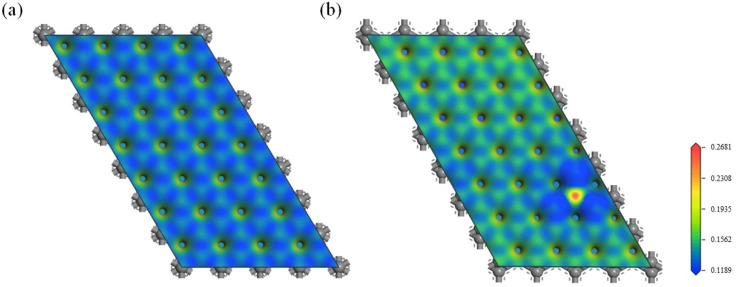
Electrostatic surface potential mapping within the (001) plane of (**a**) graphite and (**b**) boron-doped graphite across the boron atoms visualized using a chromatic scale from red (positive ESP) to blue (neutral ESP). The unit of ESP is Ha e^−1^.

The electrostatic surface potential (ESP), refering to the electrostatic potential mapped on the van der Waals surface, can be used to describe the electrophilicity or nucleophilicity of a surface. The regions with positive potentials are electron-deficient sites for nucleophilic attack. Conversely, the regions with negative potentials are electron-rich sites that preferentially interact with electrophilic reactants. The ESP-mapped van der Waals surfaces of graphite and boron-doped graphite along the (001) crystal plane are shown in Figure 7. The ESPs of both graphite and boron-doped graphite were positive and vulnerable to nucleophilic attack. The mean ESP value of the carbon layer with boron atoms shown in Figure 7b was 0.156 Ha e^−1^, higher than that of pure graphite (0.146 Ha e^−1^, listed in Table S1). The electrostatic surface potential around the boron atoms significantly increased due to the electron-deficient nature of boron, while the electron accumulation in the B-C bonds led to a decrease in the ESP at their corresponding sites. On the other hand, the overall color of Figure 7b is lighter than that of Figure 7a, implying that the global ESP value, and hence the electrophilicity, of the boron-doped graphite layer slightly increased due to the redistribution of the electrons in the Π bond and the electron-deficient nature of the carbon layer resulting from boron doping. Moreover, the global ESP value of model (a) also slightly increased, the same as that of the model (b) (Supplementary Figure S3).

#### 3.2.3. Effect of the Electron Redistribution Caused by Boron Doping on the Interlayer Spacing

**Figure 8:**
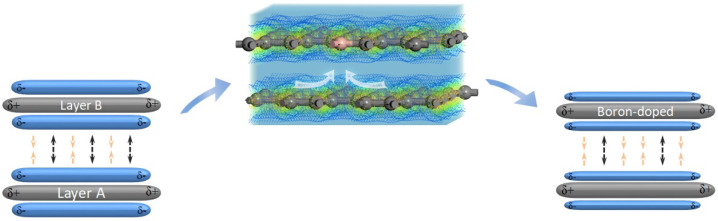
The mechanism of the effect of sub-B on the electronic structure and interlayer spacing of graphite.

Based on the analysis of the electronic structure of model (b), the mechanism of the effect of the sub-B atoms on the electronic structure and interlayer spacing of graphite is illustrated in Figure 8. As the boron atoms substituted for the carbon atoms in the graphite lattice, the electronic balance in the Π bond between the two layers was broken. Due to the electron-deficient nature of the boron atoms, 0.14 electrons were transferred from layer A (boron-free) to layer B (boron-containing), which was calculated via the analysis of the Mulliken partial charges. The electrons in the Π bond of layer A were then redistributed, which resulted in electron-deficient Π bonds in layer A. In addition, the Π electrons in layer B were also redistributed. The Π electrons around the boron atoms from layer A consequently accumulated around the carbon atoms (closest to the boron atom) because of the greater electronegativity of the carbon atoms. Due to the electron-deficient nature of the boron atoms and the electron transfer between the layers, both layers A and B were electron-deficient, leading to a decrease in the repulsion force of the Π electrons between the layers. According to our ESP analysis, the mean ESP value of the carbon layer with boron atoms (shown in Figure 7) was 0.156 Ha e^−1^, higher than that of pure graphite, because the boron–carbon layer was in an electron-deficient state. Thus, the ESPs of the electron-deficient layers A and B (shown in Figure 8) increased, resulting in an increase in their attraction to the Π electrons of the adjacent layer. Layers A and B, with high ESPs, could effectively increase their attraction to the Π electrons of the adjacent layer. Therefore, the interlayer spacing of boron-doped graphite decreased because of the increase in the attraction force between layers A and B and the Π electrons of the adjacent layer and the decrease in the repulsion force of the Π electrons between the layers. However, the repulsion forces between the nuclei and the repulsion force of the Π electrons between the layers increased when the interlayer spacing decreased, which obstructed a further reduction in the interlayer spacing until the attraction and repulsion forces between the layers reached a balance. Notably, the electrons between the layers were redistributed, and the electron density gradually increased as the interlayer spacing decreased to a value that was possibly greater than that of graphite before boron doping when the system was balanced.

## 4. Conclusions

The boron atoms that substitute for carbon atoms in graphite lattices effectively decrease the interlayer spacing. In addition, the mechanism of the effect of the substitutional boron on the interlayer spacing was revealed based on DFT calculations. According to the Mulliken partial charge analysis, boron doping broke the balance of the electrons between the layers and led to a small amount of electron transfer from an adjacent layer to a layer containing boron atoms. Due to the electron-deficient characteristic of boron atoms and the electron transfer between the layers, both the layer containing boron atoms and its adjacent layer were in an electron-deficient state, which resulted in a decrease in the repulsion of the electrons between the layers and an increase in the ESP of the layers. Correspondingly, the attractive force between the electrons in the adjacent layers (the layer containing boron atoms and its adjacent layer) increased, as determined by our ESP analysis. The increase in the attraction forces between the carbon layers and the Π electrons in the adjacent layer and the decrease in the repulsion forces of the Π electrons between the layers resulted in a decrease in the interlayer spacing. The electron density between the layers increased gradually as the interlayer spacing decreased. In short, the electron redistribution induced by boron doping played an important role in decreasing the interlayer spacing, while Poisson contraction contributed less.

## Figures and Tables

**Table 1:** Boron concentration in the samples, obtained using XPS.

Sample I.D.	B_4_C Clusters (at.%)	Sub-B (at.%)	B-O (at.%)	B (at.%)
GC27	0.32	0.45	0.13	0.90
6GC27	0.32	1.16	0.07	1.55
9GC27	0.37	1.67	0.11	2.15
3PIC27	0.27	0.54	0.13	0.94
6PIC27	0.30	0.92	0.14	1.36
9PIC27	0.32	2.00	0.20	2.52

**Table 2:** Interlayer spacings (d_002_) of the samples studied.

Sample I.D.	d_002_ (nm)	Sample I.D.	d_002_ (nm)
0GC27	0.33654	0PIC27	0.33647
3GC27	0.33557	3PIC27	0.33553
6GC27	0.33547	6PIC27	0.33546
9GC27	0.33545	9PIC27	0.33537

**Table 3:** Calculated interlayer spacings (d_002_) of pristine graphite and model (b).

Sample I.D.	d_002_ (nm)
Pristine graphite	0.3369
Model (b)	0.3323

**Table 4:** The $a_{0,G}$, $c_{0,G}$, $d_{C-B}$, $d_{C-C}$, $s_{11}\;$ and $s_{13}$ values obtained from model (b).

Sample I.D.	$a_{0,G}$	$c_{0,G}$	$d_{C-B}$	$d_{C-C}$	$s_{11}\;$	$s_{13}$
Model b	0.2459 nm	0.6738 nm	0.1485 nm	0.1412 nm	0.0009656	−0.0001537

## Data Availability

The authors confirm that the data supporting the findings of this study are available within the article.

## Supplementary Materials

The following supporting information can be downloaded at: https://www.mdpi.com/article/10.3390/ma15124203/s1, Figure S1: Mulliken partial charges of some atoms in model (a). Inset: The serial numbers of the atoms in the layers of model (a). Carbon: dark grey; boron: pink; Figure S2: Charge density diagram within the (010) plane of boron-doped graphite of model (a) across the boron atoms; Table S1: The maximum, mean and minimum value of ESPs of pure graphite and boron-doped graphite of model (b); Figure S3: Electrostatic surface potentials mapping within the (001) plane of boron-doped graphite of model (a) across the boron atoms visualized using a chromatic scheme from red (positive ESP) to blue (neutral ESP).
