# Supplementary material for: Effect of Boron Doping on the Interlayer Spacing of Graphite
Source: Materials (Basel). 2022 Jun 13;15(12):4203. doi: 10.3390/ma15124203 (PMC9229557; doi:10.3390/ma15124203)
Supplement: Supplementary file 1 [file materials-15-04203-s001.zip › materials-1750592-supplementary.pdf]

Effect of boron doping on the interlayer spacing of graphitized needle coke

Chenguang Bao [1], Qing Zeng [2], Fujin Li [3], Lei Shi [4], Wei Wu [4], Li Yang [1], Yuxi Chen [1,**], Hongbo Liu [1,*]

[1] College of Materials Science and Engineering, Hunan University, Changsha 410082, China; 18650765569@163.com (C.B.); hunanyangli@aliyun.com (L.Y.)

[2] Hunan Provincial Key Laboratory of Flexible Electronic Materials Genome Engineering, School of Physics and Electronic Sciences, Changsha University of Science and Technology, Changsha 410114, China; zengqing@stu.csust.edu.cn

[3] College of Materials Science and Engineering, Changsha University of Science & Technology, Changsha 410114, China; lifujin511@163.com

[4] Hunan Zhongke Shinzoom Co., Ltd., Changsha 410118, China; lshi@shinzoom.com (L.S.); wuwei202206@163.com (W.W.)

[*] Correspondence: yxchen@hnu.edu.cn (Y.C.); hndxlhb@163.com (H.L.); Tel.: +86-137-2386-1392 (Y.C.); +86-139-7481-7841 (H.L.)

1. Mulliken partial charges

The results of the Mulliken partial charges of atoms of model (a) are displayed in Figure S1. The total value of Mulliken partial charges around the boron atoms was -0.11, while that of the carbon atoms in the adjacent carbon layer near the boron atoms was 0.11, implying the presence of electron transfer between the layers around the boron atoms.

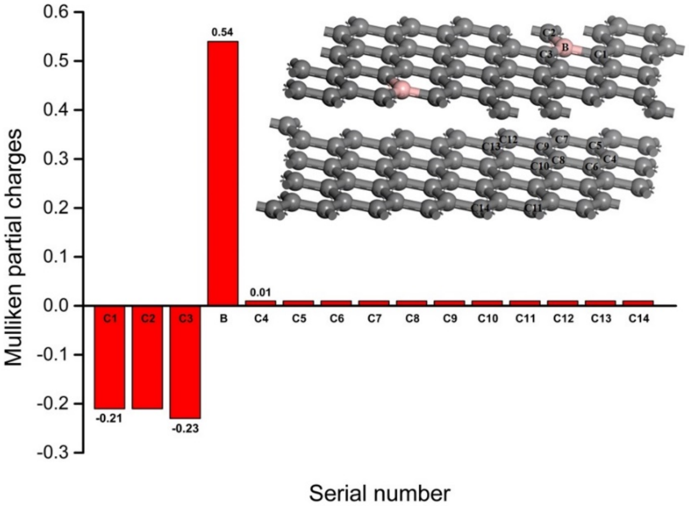

Figure S1. Mulliken partial charges of some atoms in model (a). Inset: The serial numbers of the atoms in the layers of model (a). Carbon: dark grey; boron: pink.

[*] Corresponding author. Tel: +86-13974817841. E-mail: hndxlhb@163.com (H. Liu)

[**] Corresponding author. Tel: +86-13723861392. E-mail: yxchen@hnu.edu.cn (Y. Chen)

2. Total charge density

Figure S2 shows the charge density of boron-doped graphite of model (a) along the (010) crystal plane, which can directly show the charge cloud distributed around the atoms. For model (a), the relative displacement of the layers did not occur and the charge density between the layers in the boron-doped graphite was slightly enlarged (Figure S2). The enlargement in the overlap of the Π electron cloud between the layers was the result of electron transfer between the layers and the decreased interlayer spacing.

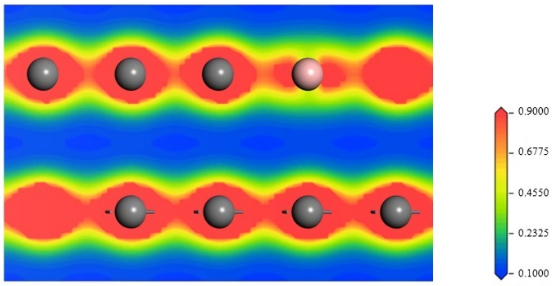

Figure S2. Charge density diagram within the (010) plane of boron-doped graphite of model (a) across the boron atoms. Carbon: dark grey, boron: pink.

3. Electrostatic surface potential (ESP)

The maximum, mean and minimum value of ESPs of pure graphite and boron-doped graphite of model (b) are listed in Table S1. The mean value of ESP of the carbon layer with boron atoms is 0.156 Ha e^{-1}, higher than that of pure graphite (0.146 Ha e^{-1}).

Table S1 The maximum, mean and minimum value of ESPs of pure graphite and boron-doped graphite of model (b) Unit: Ha e^{-1}

I.D.	Mapped maximum	Mapped mean	Mapped minimum
**Graphite**	0.204	0.146	0.119
**Model (b)**	0.289	0.156	0.112

The ESP-mapped van der Waals surfaces of boron-doped graphite of model (a) along the (001) crystal plane are shown in Figure S3. The ESPs of both graphite and boron-doped graphite were positive and vulnerable to nucleophilic attack. The global ESP value of the layer of

boron-doped graphite slightly increased due to the redistribution of the electrons in the Π bond and the electron-deficient nature of the layer resulting from boron doping, revealing an enhancement in the electrophilicity.

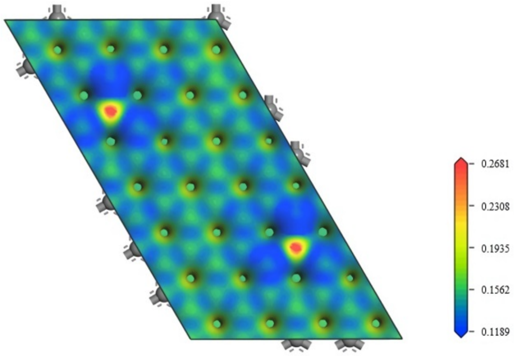

Figure S3. Electrostatic surface potentials mapping within the (001) plane of boron-doped graphite of model (a) across the boron atoms visualized using a chromatic scheme from red (positive ESP) to blue (neutral ESP).
